# Molecular Tuning of Actin Dynamics in Leukocyte Migration as Revealed by Immune-Related Actinopathies

**DOI:** 10.3389/fimmu.2021.750537

**Published:** 2021-11-15

**Authors:** Anton Kamnev, Claire Lacouture, Mathieu Fusaro, Loïc Dupré

**Affiliations:** ^1^ Ludwig Boltzmann Institute for Rare and Undiagnosed Diseases, Vienna, Austria; ^2^ Department of Dermatology, Medical University of Vienna, Vienna, Austria; ^3^ Toulouse Institute for Infectious and Inflammatory Diseases (INFINITy), INSERM, CNRS, Toulouse III Paul Sabatier University, Toulouse, France; ^4^ Laboratoire De Physique Théorique, IRSAMC, Université De Toulouse (UPS), CNRS, Toulouse, France

**Keywords:** leukocytes, cell migration, chemotaxis, actin, cytoskeleton, actin regulators, inborn errors of immunity, IEI

## Abstract

Motility is a crucial activity of immune cells allowing them to patrol tissues as they differentiate, sample or exchange information, and execute their effector functions. Although all immune cells are highly migratory, each subset is endowed with very distinct motility patterns in accordance with functional specification. Furthermore individual immune cell subsets adapt their motility behaviour to the surrounding tissue environment. This review focuses on how the generation and adaptation of diversified motility patterns in immune cells is sustained by actin cytoskeleton dynamics. In particular, we review the knowledge gained through the study of inborn errors of immunity (IEI) related to actin defects. Such pathologies are unique models that help us to uncover the contribution of individual actin regulators to the migration of immune cells in the context of their development and function.

## Introduction

Understanding how the diverse motility strategies of immune cells are controlled at the molecular level is of paramount importance when investigating immune cell responses in the context of health and disease and when designing cell-based immunotherapies. Motility is inherent to leukocyte development and differentiation for proper positioning in specific regions of lymphoid organs (1, 2). Moreover, motility is essential for mature immune cells to travel across organs and ensure their immuno-surveillance function (3, 4). Given the diversity of tissue environments and barriers crossed by any given leukocyte along its life cycle, motility needs to be regulated as a highly adaptable function (5). The intrinsic regulation of leukocyte motility relies on the integration of motility signals into adapted cell shape remodelling. This process is governed by the actin cytoskeleton that promotes the protrusive and contractile activities necessary for cell movement (6). Furthermore actin remodelling sustains other motility-related activities, such as organelle recycling, mitochondria positioning and nuclear envelope deformation (7). The molecular machinery responsible for actin remodelling comprises actin-binding proteins as well as upstream regulators accounting for a few hundreds of proteins (8, 9). In the context of leukocyte migration, the knowledge we have today about the specific roles of actin regulators stems in part from the study of rare inborn errors of immunity (IEI) caused by mutations in corresponding genes. The elucidation of molecular mechanisms underlying IEIs has revealed that more than 20 are either directly caused by, or associated with, defective actin cytoskeleton remodelling (10–14). These disease entities might therefore be considered as actinopathies specific to the immune system (from here onwards referred to as actinopathies). In this review, we present updated knowledge on actinopathies with a focus on leukocyte motility defects. In the first part of the review, we assemble knowledge on leukocyte circulation through the organism affected by actinopathies. In the second part, we turn to the cellular scale and present some of the motility challenges leukocytes face while executing their function. Finally, in the third part of the review, we zoom in on the subcellular scale and examine how actin remodelling shapes diverse cellular protrusions and ultrastructures to propel cell through dense environments following migration stimuli.

## Summary of Identified Motility Defects Across Actinopathies

**Table 1** presents an updated list of 23 actinopathies with a focus on the leukocyte motility defects characterized so far in each of these pathologies. In addition, we sorted identified defects in motility by the experimental model used in the study: primary material from patient *versus* cellular and animal models of the specific gene defect. The molecular mechanisms affected in actinopathies span multiple facets of the molecular machinery responsible for actin remodelling, as detailed in (14). Since multiple molecular layers operate upstream of actin remodelling, it is not obvious to define a threshold for the inclusion of gene defects falling under the umbrella of actinopathies. We here focus on actin itself (β-actin), actin-binding proteins or subunits of actin-binding protein complexes (ARPC1B, CORO1A, DIAPH1, HEM1, MKL1, MSN, MYH9, WASP, WDR1 and WIP), direct regulators of actin-binding proteins (CARMIL2, PSTPIP1 and STK4/MST1), RHO GTPases (CDC42, RAC2, RHOG and RHOH) and GTPase regulators (ARHGEF1, DOCK2, DOCK8, RASGRP1 and TTC7A). **Table 1** highlights crucial contribution of actinopathy discovery to our understanding of the role of individual molecular regulators in the motility and specific functions of immune cells.

**Table 1:** Actin-related inborn errors of immunity and associated leukocyte motility defects.

Actin-related inborn errors of immunity			Leukocyte motility defects	
Gene (Protein)*	Protein function	Clinical symptoms	Patient cells	Cellular and animal models
***ACTB*** (β-actin)	Non-muscle actin isoform; polymerises to F-actin	Mental retardation, recurrent bacterial and viral infections	**Neutrophils**: impaired chemotaxis in response to fMLP and zymosan-activated serum (15)	**Mice CD4+ T cells**: defective chemotaxis towards CCL21 (16)
***ARHGEF1***	GEF; regulates RhoA activity	Airway infections, defective antibody production	**B cells/T cells**: ↓ CXCL12-evoked migration (17) **T cells:** ↓ RhoA/ROCK mediated actin polymerisation upon LPA/S1P stimulation, ↓ de-adhesion on fibronectin, increased uropod length (17)	**Mice germinal centre B cells**: aberrant dissemination associated with inability to transduce S1P-evoked inhibition of migration (18)
***ARPC1B***	ARP2/3 complex subunit; polymerises F-actin branches	Failure to thrive, platelet abnormalities, eczema, infections, vasculitis, hepatosplenomegaly, thrombocytopenia	**Macrophages**: defective podosome assembly (19) **T cells**: ↓ spontaneous motility, weak IS (20), defective lamellipodium during migration and aberrant emission of filopodia-like protrusions (21) **Platelets**: defective spreading and lamellipodia assembly (22)	**THP1 cells**: defective podosome assembly (19)
***CARMIL2***	Regulates F-actin polymerisation at the barbed end	Malignancy (EBV+), IBD, recurrent skin and upper airway infections, failure to thrive	**T cells**: dispersed polarity and increased spontaneous migratory speed but ↓ directness; defective CXCL12 chemotaxis (23)	not reported**
***CDC42***	GTPase; regulates cell motility and polarity	Autoinflammation, HLH, malignant lymphoproliferation	**PBMC, BM CD34+ cells**: ↓chemotaxis toward CXCL12, abnormal filopodial pattern and cell polarization (24)	**Mice neutrophils:** ↓ neutrophil infiltration into interstitial tissues (25), loss of polarity during migration and aberrant filopodia emission instead of lamellipodium (26)
***CORO1A***	Inhibits the Arp2/3 complex; enhances F-actin disassembly *via* cofilin	Bacterial and viral infections, aggressive EBV-associated B cell lymphoproliferation, T cell lymphopenia, T-B+ SCID	**T cells**: SCID condition with visible thymus (27); severely impaired thymic output (28)	**Mice thymocytes/T cells**: impaired egress of mature thymocytes, patch-like talin-rich and abnormally distributed clusters instead of uropod (29, 30); cell-intrinsic migration defect toward SIP1, CCL21, CXCL12, defect in lymph nodes entry/egress (28) **Mice neutrophils**: defective LFA-1-dependent adhesion under flow; defective extravasation (31)
***DIAPH1***	Nucleates and elongates F-actin	Seizures, cortical blindness, microcephaly syndrome (SCBMS), mitochondrial dysfunction and immunodeficiency	**T cells**: impaired adhesion and inefficient microtubule-organizing centre repositioning to the immunologic synapse (32)	**Mice thymocytes**: ↓ chemotaxis to CCL21 and CXCL12, impaired egress from thymus (33) **Mice T cells:** impaired trafficking to secondary lymphoid organ, reduced chemotaxis (CCL21, CXCL12), ↓ production of F-actin, impaired polarity in response to chemotactic stimuli (33)
***DOCK2***	GEF; activates RAC1 and RAC2	Severe invasive bacterial and viral infections	**T, B and NK cells**: defective chemotaxis in response to CCL21 and CXCL12, ↓ actin polymerisation (34), low density of B cells, plasma cells and T cells in the lamina propria of the colon (34)	**Mice T and B cells**: ↓ motility inside T cell area and B cell follicle, ↓ S1P-induced cell migration, delayed lymphocyte egress from LN, ↓ cell motility of T cells in close proximity to efferent lymphatic vessels (35, 36)
***DOCK8***	GEF; activates CDC42	Upper airway infections, susceptibility to viral infection	**T and NK cells**: abnormally elongated shape leading to cytothripsis in confined spaces (37)	**Mice DCs:** ↓ traffic to the draining LN (38) **Mice CD4 thymocytes:** defective thymic egress and ↑ migration to CXCL12 (39) **Mice T cells:** defective transmigration and homing in LN (40) **Mice Tfh:** impaired migration to germinal centre (41) **Mice microglia**: ↓ filopodia formation (42)
***NCKAP1L*** (HEM1)	WAVE2 complex subunit; activates the ARP2/3 complex to promote branched F-actin networks	Fever, recurrent bacterial and viral skin infections, severe respiratory tract infections, poor antibody responses, autoimmune manifestations	**T cells**: defective membrane ruffling, loss of lamellipodia, reduced F-actin density at the leading edge with abnormal puncta, spikes, and blebs, ↓ migratory velocity (43, 44), lack of polarization (45) **B cells**: aberrant morphology, defective directional migration when exposed to CCL19 gradient (44) **Neutrophils:** ↓ velocity, ↓ directional persistence, misdirected competing leading edges (43), abnormal distribution of F-actinat at the leading edge instead of the lamellipodium (45)	**Mice neutrophils and macrophages**: defective migration (46, 47), spiky shape (47) and defect in actin polymerisation (46), accumulation within and near blood vessels and defective migration in 3D chemokine gradient (47) **Mice DC**: lack of lamellipodia, ↑ speed, ↑ directional persistance and migration speed paths in 3D collagen gels (48) **Zebrafish neutrophils**: defective migration (45)
***MKL1***	Regulates transcription of actin and actin cytoskeleton related genes	Severe bacterial infections, skin abscesses	**Neutrophils**: actin polymerisation defect, ↓ motility and chemotactic response, failure in firm adherence and transendothelial migration under shear flow conditions (49) **DCs**: unable to spread normally or to form podosomes (50)	**Neutrophil-like HL-60 cells**: failure of uropod retraction (50)
***MSN*** (moesin)	Links membrane proteins to actin filaments	Eczema, episodic bacterial and VZV infections, lymphopenia	**T cells**: impaired chemotaxis in response to CCL21 and CXCL12 (51)	**Mice thymocytes**: defective thymic egress (39) **Mice B cells:** defective egress from the BM (39) **Mice T cells:** defective egress from the LN, ↓ of microvilli density failed internalization of S1PR1 (52), impaired ability to exit the bloodstream (53) **Mice neutrophils:** ↑ rolling velocity in inflamed blood vessels (54)
***MYH9***	F-actin dependent motor protein	May-Hegglin anomaly, Sebastian syndrome, Fechtner syndrome, Epstein syndrome, mild macrothrombocytopenia	not reported**	**Mice neutrophils**: ↓ in migration velocity and euclidean distance during mechanotactic migration, transmigration and migration in confined 3D environments (55) **Mice T cells**: ↑ adhesion, impaired interstitial migration (56) **Human primary T cells (siRNA, blebbistatin)**: aberrant uropod elongation (57)
***PSTPIP1***	Adaptor protein; interacts with WASP	Oligoarticular pyogenic arthritis, acne, pyoderma gangrenosum-like lesions	**Macrophages**: impaired chemotaxis to M‐CSF, impaired invasion into gel, defect in podosome formation (replaced by filopodia-like protrusions) (58, 59) **CD4+ T cells**: faster motility in collagen matrix, ↑ F-actin content (60)	not reported**
***RAC2***	GTPase; regulates cell migration and polarisation	Lymphopenia, recurrent respiratory infections, poor wound healing, leukocytosis	**Neutrophils:** ↓ actin polymerisation and chemotaxis, failure to assemble lamellipodium (43, 61), defective migration to fMLP (62)	**Zebrafish T lymphoid progenitors**: inability in homing to the thymus because of defective cell-autonomous motility (63) **Zebrafish neutrophils**: impaired migration to infection site (64) **Mice T cells:** reduced chemotaxis (65) **Mice neutrophils:** decreased infiltration into interstitial tissue (66)
***RASGRP1***	GEF; activates RAS	Severe pneumonia, failure to thrive, EBV susceptibility	**CD8 T cells**: ↓ migration speed in response to CXCL12 (67)	not reported**
***RHOG***	GTPase; activates RAC1	HLH features, fever, cytopenia, low haemoglobin	not reported**	**NK-92 cells**: migration defect in response to CXCL12, CXCL13, CCL21 (68)
***RHOH***	GTPase; inhibits RAC1, RHOA & CDC42	Persistent EV-HPV infections, skin lesions	**T cells**: defect in skin-homing, ↓ percentages of T cells expressing tissue-homing markers (CLA, CCR4, CCR6, CCR10, α4β7) (69)	**HPC cells:** ↑CXCL12-induced chemotaxis and chemokinesis (70)
***STK4/MST1***	Serine-threonine protein kinase; Regulates the actin-bundling protein L-plastin	Recurrent infections, EBV infections, skin lesions and infections	**PBMCs and B cells**: defect in LFA-1-mediated adhesion and chemotaxis in response to CXCL11 (71) **T cells**: defective chemotaxis in response to CCL19 and CCL21 (72), ↓ expression of the homing receptors CCR7, CD62L (72)	**Mice thymocytes**: defect in thymic egress (73, 74), defect in response to CCL21 and CCL19 (73), and to CXCL12 and CCL25 (74) **Mice T and B cells:** impaired homing to peripheral lymph nodes and emigration from LN to blood, impaired arrest on HEV, problem in cell polarization, defective interstitial migration (73, 74) **Mice DCs:** impaired retention and/or homing of DCs in the spleen, ↓skin DC migration into draining LN (73)
***TTC7A***	Regulates the RHOA pathway	Early-onset IBD, lymphocytopenia and alopecia	**T cells**: ↑ spreading and adhesion, impaired chemotaxis toward CCL21 and CXCL12 (but ↓ receptor expression) (75)	not reported**
***WAS*** (WASP)	Activates the ARP2/3 complex to promote branched F-actin networks	Thrombocytopenia, eczema, recurrent infections, increased incidence of autoimmunity and lymphomas	**B cells**: thinner and shorter protrusions, ↓ chemotactic migration to CXCL13 (76), CXCL12 (77). reduction of area in LN (78) **DCs:** defective migration (79), unstable lamellipodia (79), fail to maintain polarization at the leading edge, inability to form podosomes, extreme elongation of uropod (80) **Monocytes:** defect in cell polarization, reduced migration to fMLP, MCP-1 and MIP-1α (81) **NK cells:** ↓ chemotactic migration and transendothelial migration toward CXCL12 and CX3CL1 (82) **Neutrophils:** impairment in integrin clustering, normal rolling but defect in arrest and firm adhesion under shear flow (83) **Macrophages:** ↓ of podosomes and abnormal polarization, ↓ number and abnormal distribution of filopodia, defective assembly of podosomes (84) **T cells:** ↓ migration in response to CXCL12 (85), depletion of lymphocytes from LN paracortical regions (78), normal localization of revertant T cells in secondary lymphoid organs (86), aberrant actin cytoskeleton dynamics at the IS (87), defective stop behaviour upon antigen encounter (88), inability to form invasive podosome, defect in trans-endothelial migration (89), disrupted lamellipodium irradiating in different directions (87, 90)	**Mice B cells:** ↓ chemotactic migration to CXCL13, CCL19, CXCL12, abnormal spleen architecture, delayed GC reaction, deficient homing to spleen and LN, aberrant microvilli formation upon anti-CD40+IL-4 stimulation (76) **Mice DCs:** no dominant leading edge, inability to detach appropriately, ↓ migration toward CCL21, delayed migration from skin to draining LN, DC abnormally retained in the MZ (91), reduced migration toward CCL3 (92) **Mice BM precursors**: impaired migration in response to CXCL12 and deficient homing (93) **Mice neutrophils:** impaired firm arrest under shear stress, ↓ migratory capacity under shear stress, delay in migration into an inflamed site *in vivo* (83) **RAW/RL5 macrophages:** ↓ of podosomes and impaired chemotactic migration to CSF1 (94) **Mice megakaryocytes:** defect in migration on CXCL12 (95) **Mice T cells**: impaired migration in response to CCL19, compromised adhesion under shear flow (96)
		X-linked neutropenia (activating mutations in WASP)	**Neutrophils,** increased migration into tissues, ↑ adhesion under shearing flow, increased in adhesion footprint and spreading area (97)	**Mice DC**: abnormal speed fluctuations and ↓ global displacement, impaired entry into the draining LN (98)
***WDR1***	Promotes severing of F-actin together with cofilin	Autoinflammation, skin and airway infections	**B cells:** defect in differentiation, no defect in CXCL12 (99) **Neutrophils**: nuclear hernations, failure to polarize in response to fMLP; impaired random and fMLP-directed migration, increased F-actin (99–101) **DCs** and **monocytes:** enlarged actin-rich podosomes, high number of podosome-like structures (99, 100)	**Mice neutrophils**: impaired chemotaxis toward MIP-2 (102)
***WIPF1*** (WIP)	Stabilizes WASP	Eczema, T cell lymphopenia and thrombocytopenia	**T cells**: impaired migration toward CCL19 and CXCL12, filamentous appearance and abnormal lamellipodium (103) **B cells:** impaired migration toward CCL19, ↓ cell speed and directional migration; emission of multipolar filopodia (103) **DCs**: defective polarization, ruffles in place of leading edge (104)	**B cell line:** unstable lamellipodium and defective directional migration in CCL19 gradient (103) **THP1 cells**: defective podosome formation and impaired transendothelial migration (105) **Mice DCs:** defective podosome formation with abnormal structure, failed to develop a major leading front and instead formed multiple simultaneous and unstable lateral lamellae and ruffles (104)

*protein name specified only when distinct from gene name.

**no data on defects in motility of immune cells in primary or animal model has been reported.

3D, three-dimensions; BM, bone marrow; CCL, Chemokine (C-C motif) ligand; CLA, cutaneous lymphocyte antigen; CSF1, colony stimulating factor 1; CXCL, C-X-C motif chemokine ligand 10; DC, dendritic cell; EBV, Epstein-Barr virus; EV-HPV, Epidermodysplasia verruciformis-human papillomavirus; fMLP, n-formyl-méthionyl-leucyl-phénylalanine; GEF, guanine exchange factor; GTPase, guanosine triphosphate hydrolysing enzyme; HL-60, human neutrophilic cell line; HLH, hemophagocytic lymphohistiocytosis; IBD, inflammatory bowel disease; IS, immune synapse; LFA-1, lymphocyte function-associated antigen 1; LN, lymph node; LPA, lysophosphatidic acid; MCP1, monocyte chemoattractant protein 1; M-CSF, Macrophage colony-stimulating factor; MIP1-α, macrophage inflammatory protein-1 alpha; MZ, marginal zone; RL-5, macrophage cell line; S1P, sphingosine 1-phosphate; S1PR1, sphingosine 1-phosphate receptor 1; SCID, severe combined immunodeficiency; THP-1, Tohoku Hospital Pediatrics-1 (human monocytic cell line); VZV, varicella zoster virus.

## Motility Defects in Actinopathies at the Organism Level

**Figure 1:**
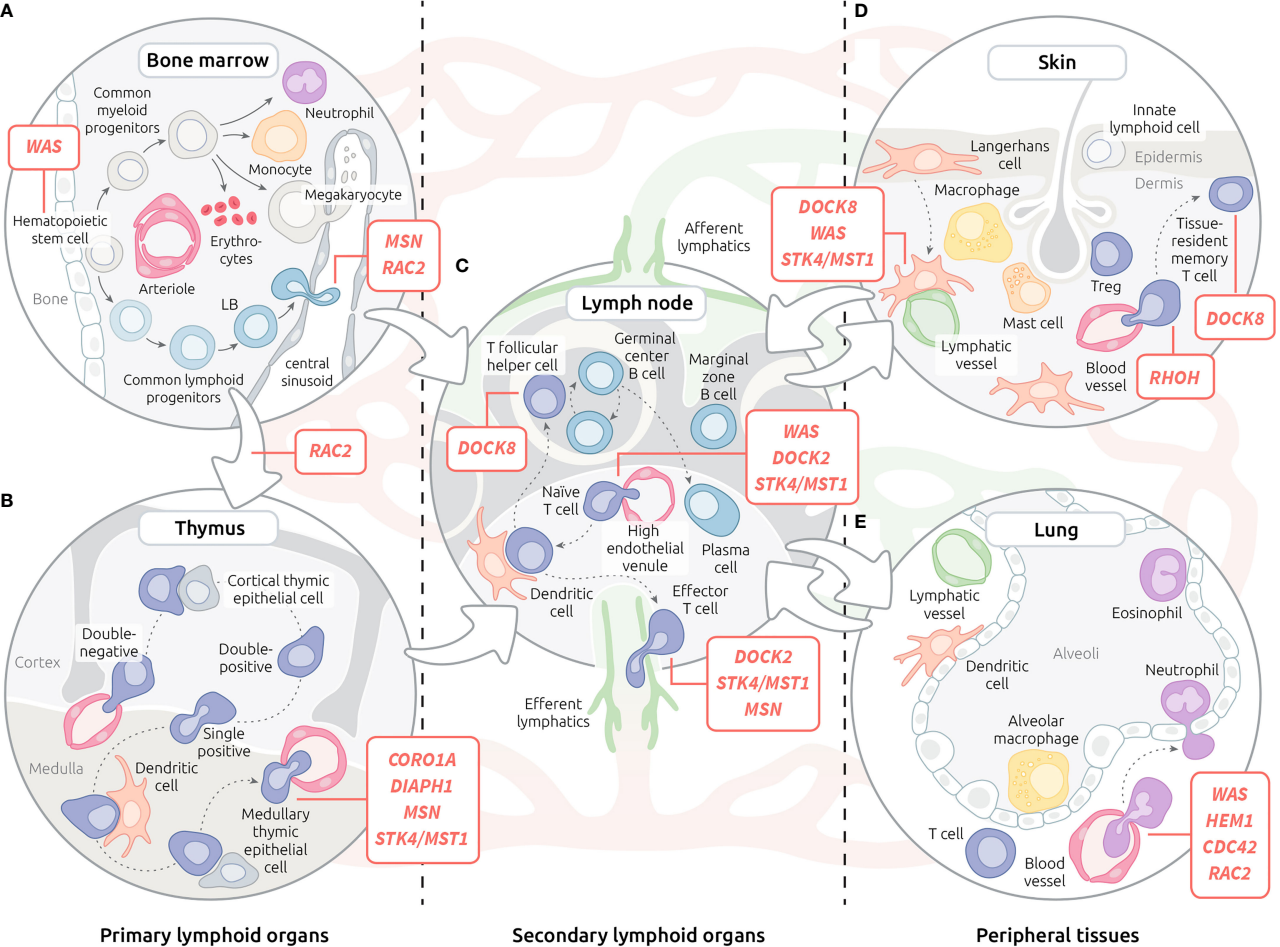
Motility defects in actinopathies at the organism level. **(A)** Leukocyte trafficking in the bone marrow. **(B)** T lymphocyte trafficking in the thymus. **(C)** Recirculation of leukocytes through blood, lymphatic system and lymph nodes. **(D)** Migration of leukocytes within the skin. **(E)** Migration of leukocytes within the lungs. Red lines indicate steps of leukocyte trafficking affected by actinopathies with affected genes displayed in corresponding bubble. Lymphatic vessels are depicted in green, while red vessels are depicted in red.

The life journey of leukocytes is indissociable from their trafficking across the organism. From their differentiation in primary lymphoid organs to their homing and recirculation in secondary lymphoid organs and peripheral tissues, immune cells navigate through various tissues (**Figure 1**). They use both blood and lymphatic systems to commute between the organs they visit or colonise. To date, most actinopathies have been found to be associated with impaired migration of immune cells within and between organs (**Figure 1**). This section will review data collected on actinopathies and complementary animal models that have provided fundamental knowledge about leukocyte trafficking at the organism scale.

### Bone Marrow Colonisation and Positioning During Hematopoiesis

The foetal liver is the initial site of hematopoiesis. After the development of bones, hematopoietic stem cells migrate to the bone marrow (BM), which then becomes the major site of maturation of most immune cells (106). The precise positioning within BM niches of developing hematopoietic cell subsets (**Figure 1A**) is important for the tuning of differentiation (107). This is controlled, at least in part, by chemokine receptors, adhesion molecules and local concentrations of Ca^2+^ and oxygen (108).

The earliest motility defect in immune cell ontogeny reported in the context of actinopathies applies to the Wiskott-Aldrich syndrome (WAS). Indeed, hematopoietic progenitors from *Was*-KO mice displayed reduced migration from foetal liver to BM (93). Impaired colonisation of BM by WASP-deficient cells could explain biased X-inactivation observed in WAS female carriers. Although the complex orchestration of hematopoietic cell positioning within the BM is expected to require motility steps and acquisition of specific motility properties as cells differentiate, little is known about the function of actin regulators in these processes. In the context of actinopathies, reported bias in peripheral blood cell counts and immunophenotype in patients may reflect defects in BM migration and positioning. Indeed, in the context of WASP deficiency, the proportion of immature B cells in the BM is decreased, while that of transitional B cells in the periphery is increased (77). Such bias in B cell development has been proposed to result from the defective ability of B cells from WAS patients to respond to CXCL12, which plays a major role in immature B cell retention in the BM.

Differently, deficiency in MSN appears to be associated with a defective egress of B cells from the BM, at least in the murine KO model (39). Interestingly, in the context of B cell development, MSN expression peaks in immature B cells. Analysis of B cell subpopulations in BM and peripheral blood point to a defective egress of immature B cells from the BM parenchyma into the sinusoids.

Deficiency in WDR1, a key actin severing protein, causes an even more severe defect in B cell differentiation (99). Indeed, patient BM displayed very low frequency of CD20^+^ B cell precursors, which was accompanied by a marked peripheral B cell lymphopenia. However, in contrast to WASP deficiency, WDR1 deficiency did not appear to affect the ability of B cells to respond to CXCL12. Rather, defective activation and regulation of apoptosis upon BCR engagement might explain the early B cell development defect in this actinopathy.

As highlighted by the Wiskott-Aldrich syndrome, proper positioning of megakaryocytes in the BM is a key step in the control of platelet production. Megakaryocytes from WASP-deficient mice displayed impaired CXCL12-evoked migration upon interaction with fibrillar collagen I (95). This combined adhesion and motility defect was shown to be associated with an inability of these cells to assemble actin-rich podosomes (see chapter 4 for detailed description). As a result of these defects, WASP-deficient megakaryocytes appeared to shed platelets ectopically within the BM space, which might explain the severe thrombocytopenia characteristic of WAS.

Beyond the few reports cited above, we currently lack insight in the relevance of actinopathy-related proteins in the multiplicity of motility steps occurring in the context of hematopoietic development in the BM. Certainly, the application of intravital imaging (109) to relevant murine models is expected to help filling this knowledge gap.

### Leukocyte Migration During Thymopoiesis

Thymopoiesis is initiated upon the migration of progenitor T cells from the BM to the thymus. Then, the negative and positive selection steps of T cell differentiation occurring in the thymus are intimately associated with regulated trafficking from cortico-medullary junction to the cortex, followed by migration to the medulla (**Figure 1B**). This trafficking is governed by several chemokine gradients and parallel up-regulation of chemokine receptors throughout the differentiation process (2). Once T cells reach the single positive stage, they initiate expression of S1PR1, the receptor of sphingosine-1 phosphate, a molecule crucial for egress from the thymus (110).

To date, only one actinopathy, RAC2 deficiency, has been suggested to be associated with a defect of migration of T cell precursors to the thymus. Depending on the effect of the mutation, RAC2-deficient patients present with severe T cell lymphopenia, suggestive of a defective thymic function (111). Interestingly, the use of a Rac2-deficient zebrafish model has revealed defective migration of T cell progenitors from the caudal hematopoietic tissue to the thymus (63), pointing to an early migration defect as the reason for T cell lymphopenia.

The migration within the thymus, in particular from the thymic cortex to the medulla area (promoted by CCL21), has been studied in CD4+ T cells from *Actb* knock-out mice. An *in vitro* transwell migration assay revealed a lack of response of these cells to CCL21, suggesting that β-actin would be necessary for this aspect of T cell motility (16).

Defect of T cell egress from thymus has been documented for CORO1A deficiency (27). CORO1A deficiency was first described in a child with a T-B^+^NK^+^ severe combined immunodeficiency (SCID) phenotype. However, unlike many SCID patients with absent or undetectable thymus, the patient had a thymic image on CT-scan, suggesting defective thymic egress rather than developmental impairment. This finding agrees with previous research of CORO1A deficiency in murine models which documented defective thymic egress as well (29, 30). This defect has been shown to be related to impaired migration of T cells toward S1P, although low survival of the CORO1A-deficient T cells might contribute to the severity of the defective thymic output (27).

In addition to CORO1A deficiency, defects in T cell egress from the thymus have been documented in murine models defective in a number of actinopathy-related molecules: DOCK8 (112), DIAPH1 (33), STK4/MST1 (73, 74) and MSN (39, 52). T cells from DOCK2-deficient mice failed to migrate toward S1P, which resulted in defective thymic egress and peripheral lymphopenia (35). The T cell lymphopenia observed in DOCK8-deficient mice was associated with accumulation of mature single positive T cells in the thymus, as a result of increased chemotaxis in response to CXCL12 (112). Knock-out of *Diaph1* in mice caused impaired chemotaxis toward CCL21 and CXCL12, associated with reduced T cell numbers in spleen and lymph node (LN), but normal cellularity and cell distribution in the thymus (33). A defective egress of *Diaph1*-/- thymocytes in response to CCL21 was identified using an organ culture of the thymus. Patients with STK4/MST1 deficiency have a profound CD4 lymphopenia with very low circulating naive CD4 and CD8 T cells. In addition, patient CD4 T cells displayed a defective migration toward CCL19 and CCL21 (72). STK4/MST1-deficient mice were shown to accumulate mature thymocytes in the thymus, which was associated with peripheral T cell lymphopenia. A transwell assay with thymic lobes was used to show that *Mst1-/-* T cells have impaired emigration from the thymus in response to CCL19 (73). In a complementary study, STK4/MST1-deficient thymocytes exhibited defective migration in response to CCL19, CCL21, CXCL12 and CCL25 but not S1P (74). Thus, STK4/MST1 could act as a signalling hub for several chemokines. Finally, *Msn*-/- mice exhibited an accumulation of mature single positive thymocytes with peripheral lymphopenia (39), in line with the finding that MSN expression is induced at the single positive stage at which it regulates the response to S1P *via* the downregulation of S1PR1 (52).

In conclusion, a number of actinopathies associated with T cell lymphopenia are associated with perturbations in the egress of mature T cells from the thymus. Whether some of the considered actinopathies might also be associated with more subtle alterations in the precise positioning of developing T cells in the different regions of the thymus remains to be investigated.

### Leukocyte Homing and Positioning in Secondary Lymphoid Organs

The architecture of secondary lymphoid organs is defined by specialized areas, the organization of which highly depends on the selective migration programs of immune cell subsets interacting in these areas (**Figure 1C**). Therefore, histological analysis of lymphoid organ biopsies in actinopathies, when available, can be informative to reveal leukocyte migration defects. This is the case for WAS patients in whom examination of LNs and spleen pointed out a reduction in T and B cell areas (78). This abnormal architecture was partly recapitulated in *Was*-KO mice that harbour reduced B cell areas and slower germinal centre reaction after immunization (76). This defect was found to be B cell intrinsic since homing capacity was impaired when WASP-deficient B cells were transferred into wild-type recipients.

Histological examination of the *lamina propria* of the colon of a child with DOCK2 deficiency suffering from colitis showed low density of B cells, plasma cells, and T cells (34). This was suggestive of a defective homing of lymphocytes to local lymphoid tissues in the context of inflammation. In agreement, data on isolated B and T cells from DOCK2-deficient patients have revealed defects in RAC1 activation, actin polymerization and migration towards chemokines. The major role of DOCK2 in driving leukocyte trafficking to the LN had previously been established in *Dock2-/-* mice (36). Indeed, the accumulation of *Dock2-/-* cells in LNs was reduced upon adoptive transfer. Multiphoton intravital microscopy has been successfully used to investigate the intra-nodal migratory behaviour of *Dock2-/-* T and B cells (35). Although these cells localized properly, they featured reduced motility with erratic oscillations in contrast to the random walk pattern observed in control cells. DOCK2-deficient T and B cells also exhibited a two-fold increase in dwelling time caused by a defect in LN egress with impaired response to S1P signalling.

DOCK8 deficiency is also associated with combined defects of lymphocyte subsets in the context of secondary lymphoid organs. Defective homing of *Dock8*-KO T cells to LNs was suggested to be attributable to the role of DOCK8 in activating WASP *via* CDC42 (40). A genetic screen revealed that DOCK8 is required for the formation of marginal zone B cells, the persistence of B cells in germinal centres and their affinity maturation. However, these B cell intrinsic defects were associated neither with homing nor with chemokine-induced motility, but rather with a defect in LFA-1 polarization at the immunological synapse (IS) (113). T cell defects in the *Dock8-*KO mice were also shown to contribute to the poor antibody responses to T cell dependent antigens. In particular the migration of T follicular helper cells to B cell follicles was severely reduced (41). The role of DOCK8 in T and B cell function is further highlighted by cases of somatic reversion showing clinical improvement as a consequence of partial functional restoration of the T and B cell memory compartments (114).

Patients with MSN deficiency typically have very low T cell count in their peripheral blood but do not experience as many severe infections as SCID patients (51). A possible explanation for this discrepancy might be an abnormal retention of MSN-deficient lymphocytes in LNs. In support of this possibility, lymphocyte entry into the spleen and LNs was documented to be only minimally impacted in the *Msn-/-* model, while lymphocytes egress from LNs was reduced (39). Such differential defect in LN entry as opposed to egress might be due to a preponderant role of MSN in regulating S1P-dependent egress, as suggested by the recent finding that ezrin-radixin-moesin (ERM) proteins are particularly important to spatially control a bleb-based motility mechanism, specifically triggered by S1P (53).

STK4/MST1 deficiency in humans is associated with reduced expression of the homing receptors CCR7 and CD62L on lymphocytes and impaired migration towards CCL19 and CCL21 (72). These defects are expected to severely impact lymphocyte homing to secondary lymphoid organs, in agreement with the reduced cellularity found in the secondary lymphoid organs of STK4/MST1-deficient mice at steady state and upon adoptive transfer of B and T cells (73). Furthermore, evidences suggest that STK4/MST1 contributes to LN and non-lymphoid tissue egress (74). In addition to its role as a transcriptional regulator, STK4/MST1 has been shown to exert a direct function on actin cytoskeleton remodelling in T cells through phosphorylation of L-plastin (115). It is therefore possible that the extended role of STK4/MST1 in regulating lymphocyte trafficking might result from its combined function as a regulator of both actin cytoskeleton and transcription.

Dendritic cells (DCs) are of critical importance for mounting an adaptive immune response. After antigen uptake in the peripheral tissue, these antigen-presenting cells migrate to the draining LNs through the lymph to activate specific T cells. It was shown in mice that WASP-deficient DCs exhibit a delayed migration from the skin to the draining LNs. In WASP-deficient animals, LNs do not increase in size and cellularity, reflecting the absence of lymphocyte traffic modification (91). Bone marrow-derived DCs were also studied in a mouse model of X-linked neutropenia (XLN), caused by gain-of-function mutations in the WAS gene (L272P). Unlike WAS-KO DC, WAS L272P DC show abnormal speed fluctuations and reduced global displacement. When tested *in vivo*, both seem to impair skin DC entry into the draining LN (98). In addition to WASP, a correct expression of DOCK8 in DCs seems to be a prerequisite for efficient T cell priming *in vivo*. Indeed, DOCK8 deficiency does not impact antigen uptake nor its presentation, but decreases DC trafficking to the draining LNs (38). A defect of skin DC migration to the draining LNs was also observed in STK4/MST1-deficient mice (73). Of note, STK4/MST1- and DOCK8-deficient patients share a common susceptibility to cutaneous warts, which could be explained by impairment of skin DC homing to the LNs.

In conclusion, a number of actinopathies are associated with alterations in the homing of lymphocytes and DCs to LNs (WASP, DOCK2, STK4/MST1) and/or in the egress of antigen-experienced lymphocytes from LNs (DOCK2, MSN, STK4/MST1). Interestingly, each examined deficiency appears to impact non-redundant mechanisms and steps accounting for LN homing and egress.

### Leukocyte Migration in Peripheral Organs

Because of their contact with the environment, the skin and the lungs represent the main sites of pathogen entry (**Figures 1D, E** **)**. It is therefore not surprising that most IEIs are associated with increased susceptibility to skin and lung infections (116). In the context of actinopathies, defective recruitment to these sites of both innate immune cells and primed lymphocytes may account for reduced ability to fight local infections.

During the primary phase of an infection, innate cells (e.g., neutrophils and monocytes) migrate to the site of inflammation. The infection spectrum observed in WAS patients may be suggestive of a neutrophil defect. This was confirmed in WASP-deficient mice where impaired integrin-dependent function in neutrophils was linked to a delay in migration into an inflamed site (83). On the contrary, despite a severe neutropenia, XLN patients are not at high risk of infections (117). This could be explained by normal numbers of neutrophils in peripheral sites, as exemplified in XLN patients saliva (97). Interestingly, neutrophils from XLN mice exhibit an increased infiltrating capacity with a competitive advantage over WT neutrophils in mixed bone marrow chimeras. The study of HEM1-null macrophages and neutrophils suggested an impaired migration *in vitro* (46, 47). More precisely, the knock-down of *nckap1l*, the gene encoding for HEM1, in zebrafish was responsible for a defective neutrophil migration after tail injury along with a decrease in circulating neutrophils (45). In a model of lipopolysaccharide (LPS)-induced lung inflammation, a prominent role of CDC42 was discovered in neutrophil emigration. Murine marrow cells with inducible KO of *Cdc42* were infused in irradiated mice. After LPS challenge, neutrophil counts in the lungs were significantly lower for *Cdc42*-/- reconstituted mice, revealing a defect in neutrophil infiltration (25). RAC2-deficient mice presented a decrease in cellular inflammatory exudate despite a persistent neutrophilic leukocytosis. This phenotype was reminiscent of leukocyte adhesion deficiency and thus suggested a defect in migration to the site of inflammation (66). To recapitulate the phenotype observed in humans, Deng and colleagues engineered a zebrafish model harbouring the inhibitory D57N mutation in Rac2 (64). This model allowed the observation of an impaired neutrophil migration to the site of infection with high neutrophil counts in peripheral blood. Interestingly, researchers discovered the role of RAC2 signalling in neutrophil retention in the bone marrow as Rac2 D57N mutation was able to partially rescue a zebrafish model of WHIM with constitutive CXCR4 signalling.

Once activated, effector T cells have to move to the site of infection (e.g., lung or skin) to exert their helper or cytolytic function. For this purpose, they express tissue homing chemokines receptors or specific integrin. Some of these T cells will acquire a tissue residency program to respond rapidly to a second antigen encounter (118). Interestingly, DOCK8-deficient patients suffer from numerous skin infections (119, 120). This peculiar infectious phenotype motivated studies on the role of DOCK8 in migration into the skin constrained environment, as we will detail in part 3. In DOCK8 deficiency, non-DC mononuclear phagocytes are prone to migration-induced cell death. This drives the skewing of the CD4^+^ T cell response to a Th2 profile in the context of respiratory tract infection with *Cryptococcus neoformans* (121). RHOH-deficient patients also display susceptibility for skin infections with persistent Epidermodysplasia verruciformis linked to human papillomavirus infections (EV-HPV), that could be explained by a defect in lymphocyte skin-homing (69). The study of RHOH-deficient patients evidenced several differences in tissue-homing markers compared to healthy donors. In particular, a defect in b7^+^ T cells was documented and confirmed in *RhoH*-/- mice.

In conclusion, the study of actinopathies has uncovered a number of key molecules driving effective trafficking and localization of immune cells within the organism. In these pathologies, defective recruitment of effector cells to tissues particularly exposed to infectious agents, such as the lungs and the skin, is at least in part accounting for the susceptibility of patients to infections.

## Motility Defects in Actinopathies at the Tissue Level

**Figure 2:**
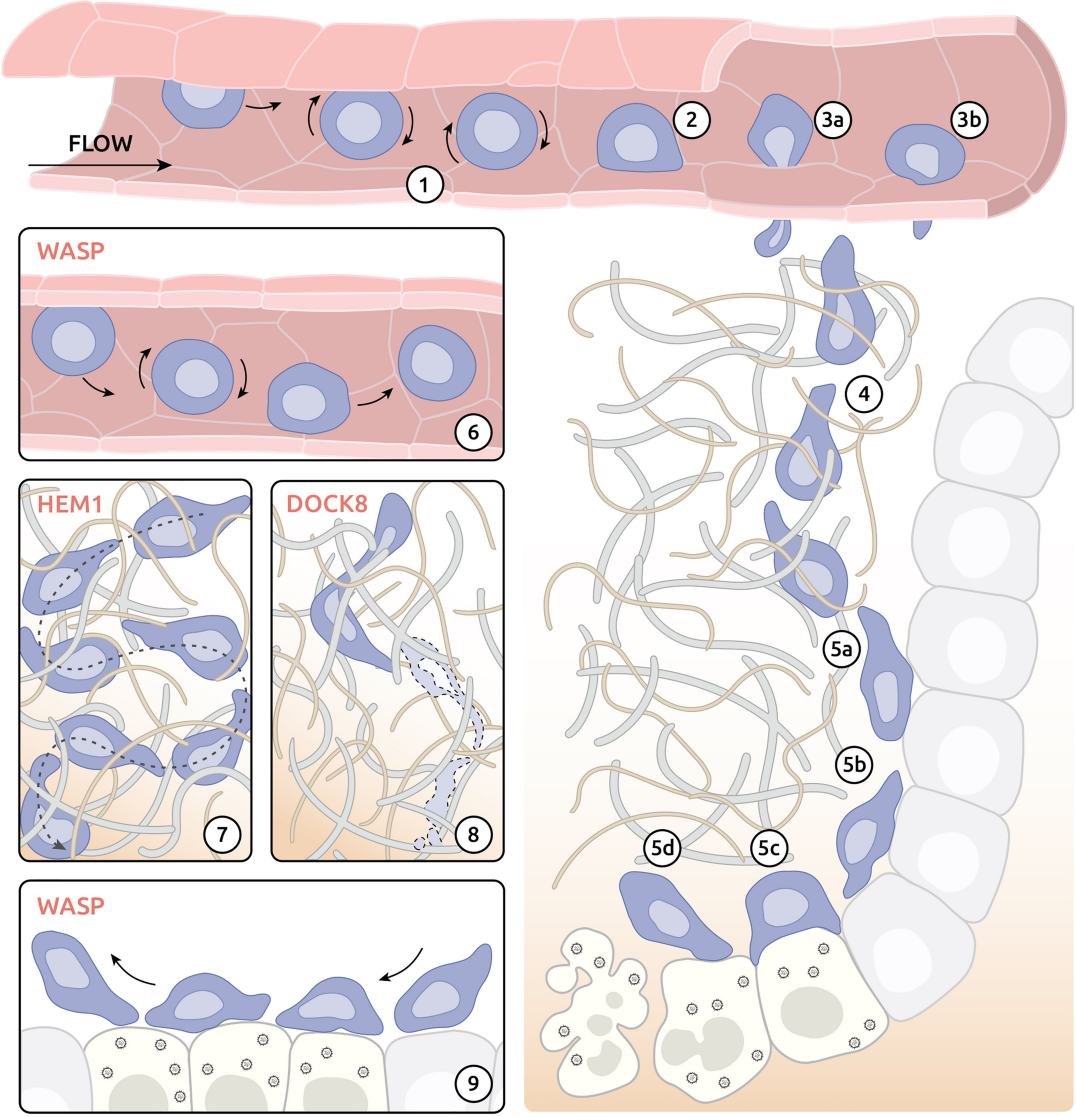
Migratory challenges and actinopathy-associated defects of effector CD8^+^ T cells on the tissue level. **(1)** Weak adhesion (rolling) of effector T cells after interaction with activated endothelium. **(2)** Adhesion at the site of exit. **(3)** Exit of the blood vessel by migration between **(3a)** or through **(3b)** endothelium cells. **(4)** Interstitial navigation following chemokine gradient. **(5)** Execution of cytolytic activity at the site of infection. **(5a)** Interaction with target cells. **(5b)** Kynapse-based scanning of target cells. **(5c)** Development of IS with infected cells and delivery of cytotoxic compounds. **(5d)** Destabilization of immune synapse (IS) and detachment from targeted cell. **(6)** Compromised attachment of WASP-deficient T cells to the endothelium at the exit site. **(7)** Reduced capacity of directional migration in tissue environment of HEM1-deficient T cells. **(8)** Cytothripsis of DOCK8-deficient T cells during migration through confined environment. **(9)** Impaired formation of IS by WASP-deficient T cells.

To gain insight into the actual leukocyte motility defects underlying actinopathies, it is important to consider the precise steps accounting for the translocation of leukocytes to tissues and their navigation within those tissues [as reviewed in (122)]. As described in chapter 2 (organism scale), among the trafficking defects associated with actinopathies are navigation of naïve lymphocytes to secondary lymphoid organs and migration of activated lymphocytes to infection sites. Although each subset of immune cells tends to migrate along a specific route, four migration steps are shared as depicted in **Figure 2**: 1) adhesion to the blood vessel at the site of priming or infection, 2) trans-endothelial migration (TEM), 3) navigation through the interstitial space and 4) interaction with a target (e.g., pathogens, antigen presenting cell or other cells of the immune system).

### Adhesion to the Endothelium

The mechanism of exit from blood circulation used by immune cells is well-studied and has been reviewed in detail elsewhere (123). In case of infection, tissue-resident sentinel cells (e.g., macrophages, dendritic and mast cells) release cytokines (including TNF-α, IL-1β or histamine), which, in turn, activate endothelial cells in proximal blood vessels. Activated endothelium increases expression of key adhesion molecules, such as selectins and integrin ligands, at its surface. Selectins are responsible for initial low-affinity adhesion of immune cells leading to rolling of immune cells along the activated endothelium (**Figure 2-1**). Rolling helps immune cells to sample chemokines bound at the surface of endothelium and eventually leads to activation of integrin receptors (e.g., LFA-1) in immune cells. Activated integrin receptors bind to endothelium with much higher affinity and allow immune cells to resist the blood flow following initial adhesion at the exit site (**Figure 2-2**).

Studies in a murine model of WAS has revealed compromised ability of WASP-deficient T cells to adhere under shear flow (**Figure 2-6**) **(** 96). Further study of XLN found that neutrophils in both XLN patients and XLN mouse models displayed dramatic increase in adhesion under shearing flow and increased migration into tissue (97). These studies suggest WASP to be important for proper adhesion of immune cells at the exit site of blood vessels. DOCK8 deficiency was also documented to result in defective attachment of T cells to ICAM-1 under flow, resulting in LN homing defects (40). In that study, DOCK8, WIP and WASP were reported to form a molecular complex positioning DOCK8 as a major guanine nucleotide-exchange factor to activate WASP.

MKL1 is a myocardin-related transcription factor, which regulates transcription of actin and multiple cytoskeleton-related genes (124, 125). Recent study of MKL1-deficient neutrophils demonstrated role of MKL1 in leukocyte adhesion under shear flow (49). Specifically, neutrophils lacking MKL1 displayed lower amount of F-actin following stimulation by an adhesive substrate.

### Trans-Endothelial Migration

**Figure 3:**
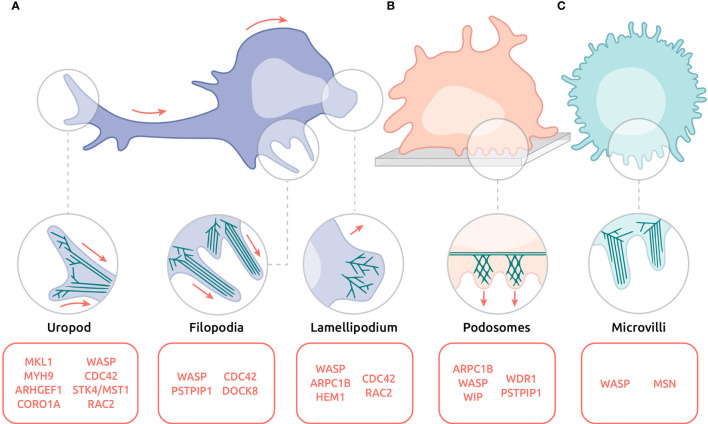
Actin-based protrusions affected in actinopathies on the ultrastructural level. **(A)** Migrating lymphocyte with uropod, filopodia and lamellipodium. **(B)** Adhering DC emitting an array of podosomes. **(C)** Circulating neutrophil decorated with microvilli. Red arrows indicate direction of movement. Protein names listed in red indicate their involvement in the corresponding protrusions.

Following arrest at the exit site, immune cells exit the blood vessel *via* the complex process of TEM. This can occur by two major mechanisms: para-cellular transmigration (**Figure 2**-**3A**) and trans-cellular transmigration (**Figure 2**-**3B**). During para-cellular TEM immune cells leave the blood vessel between endothelial cells using transient disruption of adherens junctions. In contrast, cells using trans-cellular TEM leave the blood vessel by pushing directly though endothelial cells using invasive podosomes (89). By probing the physical properties of the endothelium, leukocytes may opt for the path of least resistance, as shown by manipulating junctional integrity (126). Breaching of endothelial cell junctions or foraging through endothelial cells require protrusive activities and high deformability, both coordinated by actin remodelling (127).

Seminal study of trans-cellular TEM mechanism revealed a key role of WASP in T cell migration (89). Researchers demonstrated that invasive podosomes (reviewed in detail in chapter 4) are crucial for trans-cellular TEM of T cells. Furthermore, results showed that WASP-deficient lymphocytes were unable to form invasive podosomes and failed to migrate through endothelial cells by trans-cellular TEM. These findings suggest that immune cells lacking WASP are restricted to para-cellular TEM to exit blood vessel. It is, however, unclear if compromised adhesion of WASP-deficient immune cells under shear flow discussed in chapter 3.1 is linked to inability of these cells to form podosomes.

In addition to WASP deficiency, studies of immune cells from patients deficient in proline-serine-threonine phosphatase interacting protein 1 (PSTPIP1), a scaffolding protein involved in the regulation of WASP activity (128, 129), showed defective podosome formation as well (58). Further experiments would be required to clarify whether PSTPIP1 deficiency compromises TEM *in vivo*.

Recently, 3 additional actin-related deficiencies have been added to the list of actinopathies affecting TEM of immune cells: MKL1 (49), HEM1 (47) and ERM (53) deficiencies. MKL1 deficiency led to poor adhesion and TEM of patient neutrophils (49). Studies of HEM1 deficiency in murine models showed drastic accumulation of HEM1-deficient myeloid cells both within and near the blood vessels (47). It is unclear, however, which step of immune cell migration (trans-endothelial or interstitial) might be affected in HEM1-deficient cells. Finally, recent study of ERM-deficient mice revealed crucial role of proper coupling between actin cytoskeleton and plasma membrane in TEM of murine T cells (53). Specifically authors showed impaired ability of T cells from MSN and moesin/ezrin-deficient mice to exit the blood stream.

### Interstitial Migration

After crossing the endothelial barrier, leukocytes migrate to their targets through interstitium and tissue environments (**Figure 2-4**). Immune cells may employ two major modes of migration: adhesion-dependent and adhesion-independent [reviewed in (122)]. Both modes of migration are extremely dependent on active and precise remodelling of actin cytoskeleton but differ in mechanism of action. Adhesion-dependent migration involves attachment of plasma membrane receptors at the leading edge (such as integrins) to the extracellular substrate followed by crosslinking of membrane receptors and actin cytoskeleton. Resulting link of extracellular matrix to actin cytoskeleton allows cells to use a molecular clutch mechanism for propulsion of the cell body forward (130, 131). In contrast, adhesion-independent migration uses combination of reward actin flow and contractility of actomyosin cortex to match the topology of surrounding 3D environment. Such mechanism allows for a “chimneying” type of directional motion that applies to lymphocytes, neutrophils and DCs (132). Although adhesion-dependent and adhesion-independent migration modalities have classically been opposed, leukocytes appear to be able to use a continuum of strategies, as shown by the use of channels with variable geometries (132).

To date, studies of virtually all actin-related IEIs revealed defects in immune cell migration *in vitro* (**Table 1**). In contrast, data on ability of immune cells to migrate *in vivo* is available for a handful of gene defects: *DOCK8* (37), *DOCK2* (35), *NCKAP1L* (47) and *MYH9* (55). Deficiency of DOCK8, an atypical immune system specific guanine nucleotide-exchange factor, is probably one of the best described actin-related IEIs affecting interstitial migration of immune cells. *Ex vivo* studies of DOCK8-deficient T cells migrating in the skin revealed dramatic stretching of the cell leading to cell death by stretching-induced rupture termed cytothripsis (37) (**Figure 2-8**). The exact mechanism of DOCK8 deficiency induced cell rupture remains to be clarified. Interestingly, studies of other DOCK deficiencies (e.g., DOCK2) found normal interstitial migration of immune cells (35). Specifically, lymphocytes from DOCK2-null mice successfully navigated through complex 3D environment albeit with decreased velocity.

Recent studies of HEM1 (47)- and MYH9 (55)- deficient cells expanded the number of actin-related IEIs affecting interstitial migration of immune cells. Mouse models of both HEM1 and MYH9 deficiencies demonstrated compromised ability of leukocytes to migrate in a 3D chemokine gradient (**Figure 2-7**). Directionality of immune cell migration, however, was preserved in both deficiencies.

### Dynamic Assembly of the Immunological Synapse

The immunological synapse (IS) describes a tight junction formed by a T cell with either an antigen-presenting cell or an infected cell. At the tissue level formation of IS consists of 4 major phases (**Figures 2-5 A~D**): a) initial contact, b) spreading, c) mature synapse and d) destabilisation and termination [reviewed in (14)]. All phases of IS formation are controlled by a complex network of activatory and inhibitory stimuli and require sophisticated orchestration of actin-cytoskeleton [reviewed in (133)]. In accordance, numerous actin-related IEIs have been associated with defects in morphology and functions of IS, such as T cell activation, cytokine secretion and cytolytic activity (reviewed in (14); summarized in **Table 1**). In addition to T cells, the other subsets of lymphocytes including B cells, NK cells and innate lymphoid cells (ILCs) also assemble IS with partner cells or target cells. Although the molecular composition of the IS might vary among those lymphocyte subsets, its dependence on actin remodelling is a shared property. Exemplified by WASP deficiency, it is therefore expected that IS defects across numerous lymphocyte subsets are associated with numerous actinopathies.

To date, little is known about the impact of actin-related IEIs on interaction dynamics between lymphocytes and their targets in the context of tissue environments. One may speculate that deficiencies in WASP and DOCK2 for example might alter lymphocyte scanning activity. Indeed the roles of WASP in the stabilization and termination of the IS (88, 134–136) are expected to translate into biased interaction of lymphocytes with antigen-presenting cells or target cells in peripheral organs (**Figure 2-9**). The over-stabilization of the IS observed between CD4^+^ T cells and DCs in the DOCK2-deficient mice (137) is also expected to alter the serial scanning behaviour of the CD4^+^ T cells. More *in vivo* studies however, would be needed to clarify the impact of the actinopathy-related proteins to the motility behaviour of various lymphocyte subsets in the context of antigen search and IS dynamics.

## Motility Defects in Actinopathies at the Ultrastructural Level

The highly adaptable motility property of leukocytes is supported by their ability to emit a variety of highly dynamic actin-rich protrusions (**Figure 3**). Protrusions such as leading-edge lamellipodia and filopodia may exert an exploratory function. Others like podosomes and microvilli may rather promote and regulate adhesion. The uropod at the trailing edge is the site of contractile activities that regulates cell detachment in the context of motility. In addition, the actin cytoskeleton is involved in controlling the squeezing of the nucleus in the context of migration through confined spaces. Section below summarizes cellular protrusions affected in actinopathies. These natural deficiencies thereby highlight the key function of actin regulators in controlling leukocyte motility at the ultrastructural level.

### Lamellipodium

Located at the leading edge, the lamellipodium is a large expansion of the plasma membrane, generated by actin polymerization and branching **(** **Figure 3A-Lamellipodium** **)** [see (138) for review]. In lymphocytes it supports not only the exploratory behaviour during migration but also IS assembly during APC encounter (139). In accordance with the major role of actin cytoskeleton remodelling in lamellipodium formation, defects in its development and organization have been reported in deficiencies affecting the ARP2/3 complex-dependent process of actin branching. ARPC1B is an essential subunit of the ARP2/3 complex that is critical for its assembly and maintenance (140). Upon interaction with a surface coated with ICAM-1 and α-CD3 antibodies to mimic APC encounter, ARPC1B-deficient T cells fail to assemble a circular lamellipodium and instead emit aberrant thin filopodia (20, 21, 90). ARPC1B deficiency in human T cells is also associated with defective migration in response to chemokines (20), presumably because of a defective lamellipodium. Indeed, ARP3 subunit knock-down, which also leads to a destabilization of the ARP2/3 complex, results in a reduced exploratory behaviour of murine T cells in both 3D collagen matrices and zebrafish embryos (141). Interestingly, reduced motility of ARP3-deficient T cells is associated with reduced cortical tension and a switch from the leading-edge lamellipodia to blebs.

Although WASP operates as an ARP2/3 activator, its involvement in the regulation of lamellipodial protrusion is not as prominent as it is for ARP2/3 (142, 143). Indeed, WASP is not essential for lamellipodium emergence, but rather controls its stability and dynamics, as shown in T cells in the context of IS assembly (134, 135). Instead of emitting a radially distributed lamellipodium, WASP-deficient CD8^+^ T cells display a disrupted lamellipodium that irradiates in different directions (87, 90). Alternation of arrest phases at the contact with APC and motility phases to search for new APC is key to the tuning of lymphocyte activation and function. In this context WASP appears to play a particularly central role since its ubiquitination-dependent degradation activated upon TCR ligation sets the turnover of the IS (136). In line with the role of WASP in stabilizing the lamellipodia of T cells and DCs isolated from WAS patients emitted unstable lamellipodia (79) and failed to maintain polarization at the leading edge (80). On the other hand, a study of neutrophils carrying a gain-of-function mutation in WASP (L270P) showed an increase of the spreading area of the lamellipodia compared with normal neutrophils, which was correlated with an increase in the adhesion footprint (97).

In agreement with its function as a chaperone of WASP (144), WIP plays a prominent role in lamellipodial assembly as well. Upon interaction with surfaces coated with ICAM-1 and α-CD3 antibodies or fibronectin and chemokines, T cells derived from a WIP-deficient patient failed to assemble lamellipodia (103). Furthermore, upon exposure to a CCL19 gradient, WIP-depleted B cells emitted multipolar filopodia and pseudopodia, which were associated with reduced speed and directional migration (103). Comparably, WIP-deficient DCs displayed defective polarization due to unstable lamellae and ruffles in place of the leading edge (104).

The pentameric WAVE complex is a key ARP2/3 activator in addition to WASP, albeit sustaining distinct cellular activities (61, 145, 146). HEM1 is a hematopoietic-specific subunit of the WAVE complex. Recent studies found deficiency in HEM1 to be a novel form of immune-related actinopathy (43–45). In the absence of HEM1 the other subunits of the WAVE complex are unstable and degraded. Deficiency of the WAVE complex severely affects actin branching and consequently lamellipodial dynamics. HEM1-deficient T cells struggle to spread and lack lamellipodial protrusions. In addition, HEM1 deficiency leads to a decrease in F-actin density at the leading edge (43, 44). Upon stimulation with ICAM-1, HEM1-deficient T cells isolated from patients, present lack of a leading edge or an abnormal distribution of F-actin at the leading edge instead of the lamellipodium (45). HEM1-deficient B cells also displayed an aberrant morphology associated with defective directional migration when exposed to a CCL19 gradient (44). As reported above for ARP3 knock-down, HEM1-deficient B cells adopted a bleb-driven type of motility. Before the discovery of HEM1 deficiency in humans, its role had been studied in the context of murine DC migration (48). Interestingly, the lack of lamellipodia in *Nckap1l*-/- immature DCs was associated with increased speed and unusually straight migration paths in 3D collagen gels. Preserved motility of HEM1-deficient cells suggests that the lamellipodium of leukocytes acts as an exploratory device allowing change in direction rather than as a force generating structure (48). This notion is sustained by TIRF analysis of the actin cytoskeleton in migratory DCs, neutrophils, T and B cells which showed that the lamellipodium undulates at the front of the cells and makes only transient and limited contacts with the substrate (48, 147). A recent study widened the role of HEM1 in supporting lamellipodia assembly in macrophages and platelets. In particular, HEM1-deficient macrophages adopted a spiky shape with filopodia emitted instead of lamellipodia, which was associated with reduced motility in collagen gels (47). Taken together, studies on HEM1 deficiency indicate that the WAVE complex is a crucial driver of ARP2/3-dependent lamellipodia assembly in hematopoietic cells.

The RHO GTPases RAC1 and RAC2 act as upstream regulators of lamellipodia assembly by activating the WAVE complex. Studies of patients suffering from severe disease associated predominantly with neutrophil dysfunction identified dominant negative mutations in RAC2, altering the activity of both RAC2 and RAC1 (148, 149). Upon stimulation with fMLP, patient-derived neutrophils failed to assemble a lamellipodium and to polarize. Similarly, neutrophils with mutated RAC2 failed to migrate in response to zymosan-activated serum. Study of a loss-of-function mutation in RAC2 in patients suffering from common variable immunodeficiency revealed impaired chemotaxis of RAC2-deficient neutrophils (150). Preserved activity of RAC1 in these patients could result in residual neutrophil function and, thus, explain the distinct clinical phenotype of RAC2 deficiency. Furthermore, recent study of 3 patients with a gain-of-function mutation in RAC2 showed defective migration of neutrophils to fMLP as well (62).

### Filopodia

Filopodia are usually described as thin protrusions, composed of bundles of parallel actin filaments, that emerge from the tip of the lamellipodia of migrating cells **(** **Figure 3A-Filopodia** **)** [see (151) for review]. Lamellipodia-associated filopodia are considered as sensors. In leukocytes, filopodia are more often dissociated from the lamellipodia and may emerge as micrometre-long protrusions from different parts of the cell body. In addition to their role as environment sensors, filopodia play a role in phagocytosis in macrophages (152).

CDC42 is considered the main RHO GTPase involved in filopodia assembly (153). Recent study of the distinct immunological syndrome with immune dysregulation and inflammation identified mutation in CDC42 (R186C) leading to impaired ability of the protein to interact with its binding partners: IQGAP1 and, to a lesser extent, WASP (24). In the affected patients, both fibroblasts and immune cells displayed lack of cell polarization associated with aberrant density and distribution of filopodia. In addition, hematopoietic stem cells, peripheral blood mononuclear cells (PBMCs) and NK cells from affected patients displayed reduced migratory capacity towards CXCL12.

An early study on the CDC42 activator DOCK8 (42), showed that disruption of actin polymerisation induced by Cytochalasin D treatment of mice microglia led to accumulation of DOCK8 at the sites of filopodia formation. However, similar treatment of cells from DOCK8-deficient mice led to a 30% reduction in number of cells with filopodia compared to cells from WT mice.

As a further support of the role of the CDC42 axis in regulating filopodia assembly and dynamics, WASP deficiency was reported to result in a decrease in the number of filopodia in macrophages. The remaining filopodia occupied a large distribution around the cell instead of being concentrated in one specific edge (84). Moreover, a reduction of the length of filopodia-type protrusions in B cells has been observed in the context of WASP deficiency (76).

### Uropod

The uropod is a highly contractile structure at the rear of migrating cells, and plays a decisive role in cellular migration [see (154) for review] **(** **Figure 3A-Uropod** **).** The cell propulsion relies on myosin-based contractions at the uropod that allow detachment from the substrate and rearward squeezing (155).

A number of studies in cell lines and murine models have assessed the role of MYH9 in leukocyte motility. A study on human lymphocytes observed that the uropod of migrating T cells is enriched in MYH9 (57). Chemical inhibition of MYH9 activity with blebbistatin and genetic inhibition with siRNA resulted in aberrant elongation of the uropod, suggesting that the de-adhesion process was impaired upon the loss of MYH9 activity. Other studies showed that T lymphocytes can rapidly switch between an adhesion-dependent sliding motility and an amoeboid walking motility that depends on MyoIIA (156). *Myh9* conditional knockout in T cells impaired the turnover of adhesion sites, resulting in increased adhesion and impaired interstitial migration *in vivo* (56). Interestingly, a downregulation of MYH9 in mice neutrophils showed no defect in term of cellular shape (with a rear comparable to a normal uropod), but caused decrease in migration (55). *MYH9* mutations in humans have been found to be the cause of heterogeneous group of diseases, mostly characterized by macrothrombocytopenia, hearing loss and renal disease (157). Whether defects in the motility of leukocytes and in particular uropod dynamics might contribute to yet uncharacterized immune cell defects in individuals with *MYH9* mutations remains to be investigated.

Although CDC42 is mainly localized at the cell leading edge, it is also involved in the modulation of the myosin light-chain pathway at the uropod (26). *Via* its control over WASP activation, CDC42 has also been reported to regulate CD11b reorganization at the uropod (25). In line with a role of the CDC42-WASP axis in uropod dynamics, WASP-deficient leukocytes displayed extreme elongation of uropods (80). Abnormally elongated uropods in T cells have also been described in the context of deficiency in ARHGEF1, a regulator of RHOA GTPase. This defect was associated with a decrease in the mean square displacement of migrating T cells (17). A failure of uropod retraction was also reported in MKL1-deficient neutrophil-like HL-60 cells, presumably because of a reduced expression of myosin light chain 9 (MYL9), a component of the myosin II complex (50). Deficiency in CORO1A in mice has also been reported to impair uropod assembly in T cells. Instead of this structure, CORO1A-deficient T cells formed several patch-like talin-rich clusters, abnormally distributed around the cell cortex (30). The uropod has also been reported to be affected in RAC2 deficiency (148) and STK4/MST1 deficiency (73), highlighting the complexity of the molecular control of actin dynamics in the context of this cellular structure.

### Podosomes

Podosomes are actin-driven micron-scale cellular adhesive protrusions and are crucial for adhesion, migration and degradation of the extracellular matrix. The actin cytoskeleton within podosomes is mostly composed of branched actin filaments. Moreover, podosomes are linked together by a network of unbranched actin filaments [see (158) for review] (**Figure 3B-Podosomes**).

In this context, the WASP-ARP2/3 pathway appears crucial in the assembly of podosomes, while the WAVE complex is dispensable. Indeed both WASP and ARPC1B deficiencies are associated with the defective assembly of podosomes by monocyte-derived macrophages (19, 84). In contrast, macrophages from *Nckap1l*-KO mice have a preserved ability to assemble podosomes although they display multiple defects in lamellipodia, focal adhesions and endocytosis (47). The specificity of WASP in activating ARP2/3 towards podosome assembly might be related to its specific location in podosome priming areas and its ability to recruit ARP2/3 at such sites (84). Reconstitution experiments in WAS patient-derived macrophages using micro-injections of full-length human WASP sequence suggested that the chemotactic response to the cytokine colony stimulating factor-1 is dependent on WASP-driven assembly of podosomes (159). To date, studies of WASP revealed its key role in podosome assembly in multiple cellular systems beyond macrophages: immature DCs (80), THP-1 (19) and T cells (89). Moreover, experiments using RNA interference to induce partial down-regulation of the intracellular WASP level in DCs led to compromised podosome formation (160), indicating that a precise regulation of WASP expression might be crucial for assembly of podosomes.

As an essential partner of WASP, WIP has been reported to be essential for podosome assembly as well. In WIP-deficient mice, DCs formed few podosomes associated with loss of podosomal structure, including the actin core and the vinculin rings (104). Moreover, the authors found that lack of podosome formation in WIP-deficient DCs is compensated by assembly of large vinculin-rich focal adhesion contacts. In human macrophages, WIP localizes to the core of podosomes, where it co-localizes with F-actin (105). To date, however, defects in podosome formation have not been examined in WIP-deficient patient cells.

Studies of patients with mutations in *PSTPIP1* found decreased numbers of macrophages emitting podosomes as well (58). Similar to WASP-deficient cells, deficiency in PSTPIP1 led to increase in the number and the size of vinculin-containing focal complexes compared to normal cells. A follow-up study proposed that defect of migration of PSTPIP1-deficient macrophages resulted in a switch from formation of podosomes to filopodia (59).

MKL1 deficiency is associated with a severe defect in podosome formation resulting in impaired adhesion (50). Indeed, a complete absence of podosomes has been reported in MKL1-deficient DCs on fibronectin. Conjointly, the spreading of MKL1-deficient cells was reduced, and the F-actin staining was severely decreased compare to normal DCs.

As opposed to deficiencies in ARPC1B, WASP, WIP, PSTPIP1, and MKL1, deficiency in WDR1 is associated with a reinforcement of podosomes in myeloid cells (100). WDR1, together with cofilin, promotes actin severing and is important for actin filament turnover. Its impact on leukocyte podosomes has been initially described on monocyte-derived DCs (100). In these cells, the number of podosomes was close to that of normal cells, but their volume and F-actin content were increased. A complementary study on WDR1-deficient monocytes on fibronectin found an abnormally high number of actin-rich podosome-like structures compared to normal monocytes (99). These findings support the notion that WDR1-mediated actin filament turnover is particularly important for podosome formation.

### Microvilli

Microvilli are very thin finger-like protrusions that tend to decorate most of the cell surface **(** **Figure 3C-Microvilli** **).** In leukocytes, they have initially been described by scanning electron microscopy in B and T cells (161), macrophages (162) and DCs (163). Their function in leukocytes is to help migration and assist antigen sensing [see (164) for review]. In particular, in T cells most of the TCR molecules are localized at the surface of the microvilli, increasing the probability to detect antigen (165).

Initial studies have reported WASP as a key regulator of microvilli. Lymphocytes from WAS patients were shown to harbour reduced density of what appeared as blunted microvilli (166, 167). However, in a more recent study microvilli density was found to be unaltered in fresh WASP-deficient human lymphocytes (168). Furthermore, *Was-*KO B cells were observed to have aberrant microvilli formation upon anti-CD40 plus IL-4 stimulation, but not upon LPS stimulation (76). These studies suggest that the WASP-dependent assembly of microvilli in lymphocytes is dependent on the activation status of these cells. In addition to WASP, MSN appear to also regulate microvilli assembly. Indeed, experiments using scanning electron microscopy on murine MSN-deficient lymphocytes revealed a decrease of microvilli density, as well as defective development of these structures (39).

### Nucleus and Organelles

Deformability is an essential property of leukocytes that allows probing narrow interstices and migrating through very constrained environments. Whereas the plasma membrane is extremely deformable, the nucleus is more rigid, thereby imposing a physical checkpoint for leukocyte motility. The squeezing of the nucleus is well described in multiple cell types [see (169–171) for reviews]. In leukocytes, this process is associated with an active mechanism of pore-size discrimination facilitated by frontward positioning of the nucleus (172). Indeed, to facilitate crossing of the endothelial barrier, T cells use MyoIIA-driven contractility to squeeze the nucleus through the endothelial junctions (173). In addition, a formin-dependent actin polymerization mechanism was shown to push the nucleus at the back of effector T cells migrating in constrained inflamed tissues (174). In particular, FMNL1 was shown to promote actin polymerization at the back of the cell to enable translation of the rigid nucleus through restrictive barriers of extracellular matrix. DIAPH1, a formin highly expressed in leukocytes, has recently been shown to be associated to immune cell defects in humans when mutated (32). Whether DIAPH1 also contributes to nucleus squeezing has not yet been investigated. A further striking evidence for the role of actin remodelling in the integrity of the nucleus in the context of leukocyte migration stems from the DOCK8 deficiency. As described in chapter 3, DOCK8-deficient T cells migrating in dense environments display abnormally elongated shapes leading to cytothripsis. Interestingly, pieces of deformed nuclei were observed in the cellular fragments resulting from this atypical cell death process. This suggests that DOCK8 plays a key role in nucleus integrity by regulating the mechanical forces imposed on migrating T cells (37).

Multiple aspects of intracellular vesicle trafficking are sustaining cell polarity and motility (175). These mechanisms, such as endo- and exocytic pathways, are increasingly recognized as being dependent on vesicle-associated actin remodelling activities (176).

In the context of actin-related IEI, we currently lack direct evidence that the involved molecules might control immune cell motility *via* the regulation of vesicle trafficking.

However, given the fact that proteins such as WASP regulate endosome trafficking (177), it would be interesting to further investigate the possible contribution of vesicle trafficking to the actinopathy-associated cell motility defects.

## Outlook

The generalization of exome sequencing as an approach to identify the genetic aetiology of rare IEIs has led to the recent characterization of multiple actinopathies. This review highlights 23 gene defects related to actin cytoskeleton remodelling in immune cells and highlighted the motility impairments associated with these defects. Although alteration of leukocyte motility is not the sole explanation for the complex immune dysfunction associated with actinopathies, it certainly plays a major role. Leukocyte motility, when viewed through the prism of actinopathies highlights gaps in our knowledge of molecules and pathways crucial for the integrity of the immune system. Many actinopathies share defects in leukocyte motility (e.g., T cell egress from the thymus, homing of lymphocytes to secondary lymphoid organs, migration towards chemokines, assembly of the uropod of migrating lymphocytes) while others display unique defects specific to certain molecules (e.g., cytothripsis in DOCK8-deficient T cells during interstitial migration). Partial overlap of defects in immune cell motility among different actinopathies suggests that multiple leukocyte motility steps require the combination of multiple actin remodelling activities.

Research on actinopathies provided unique insight into the regulation of leukocyte motility. However, our current picture of the role of individual disease-related molecules remains very fragmented. Lack of detailed understanding is related to both novelty of many of the deficiencies and the amount of work it takes to apprehend the multiplicity of motility steps that immune cells undergo. Moreover, studies of immune cell motility are further complicated by the diversification of motility strategies adopted by the different immune cell subsets and restricted amount of patient material available. In an effort to address some of those limitations we have recently introduced morphological profiling of leukocytes *via* high-content cell imaging (90). Such approach can be applied to restricted sample sizes, can be used in standardizing the comparison of morphological defects in primary leukocytes from patients and has proven to be efficient at discriminating defects in closely related deficiencies such as ARPC1B and WASP deficiencies. Beyond high-content cell imaging, recent advances in super-resolution microscopy and design of probes and micro-patterned surfaces for live imaging are expected to probe cell migration more precisely and identify novel actinopathy-related leukocyte defects. Furthermore, a more systematic application of live tissue imaging in murine or zebrafish models is expected to complement *in vitro* studies with patient cells and fill the gap in our understanding of leukocyte motility across the different cellular and tissue scales.

## Author Contributions

AK, MF, CL, and LD conceptualized the review and the figures. All authors contributed substantially to the text of the manuscript. All authors approved the submitted version.

## Funding

This work received support from WWTF (PrecisePID project LS16-060 to LD) and CNRS (International Research Project SysTact to LD). MF is supported by the Fondation pour la recherche médicale (FDM202006011216).

## Conflict of Interest

The authors declare that the research was conducted in the absence of any commercial or financial relationships that could be construed as a potential conflict of interest.

## Publisher’s Note

All claims expressed in this article are solely those of the authors and do not necessarily represent those of their affiliated organizations, or those of the publisher, the editors and the reviewers. Any product that may be evaluated in this article, or claim that may be made by its manufacturer, is not guaranteed or endorsed by the publisher.
